# Incidence of and Risk Factors for Post-Operative Urinary Retention Following Surgery for Perineal Tears Among Ugandan Women: A Prospective Cohort Study

**DOI:** 10.1007/s00192-024-05855-8

**Published:** 2024-07-10

**Authors:** Musa Kayondo, Onesmus Byamukama, Brenda Ainomugisha, Rogers Kajabwangu, Paul Kato Kalyebara, Leevan Tibaijuka, Henry Mark Lugobe, Verena Geissbühler

**Affiliations:** 1 Faculty of Medicine, Mbarara University of Science and Technology, P.O.BOX 1410, Mbarara, Uganda; 2 Department of Obstetrics and Gynecology, Mbarara Regional Referral Hospital, P.O.BOX 40, Mbarara, Uganda; 3 Department of Gynecology, University of Basel, Basel, Switzerland

**Keywords:** Perineal tears, Post-operative urinary retention, Risk factors, Surgery, Post-void residuals

## Abstract

**Introduction and Hypothesis:**

We aimed to determine the incidence and risk factors for post-operative urinary retention (POUR) following surgery for perineal tears, and to determine the time to normal voiding after POUR.

**Methods:**

This was a prospective cohort study of women who underwent surgery for old (≥ 3 months) obstetric perineal tears from January 2022 to December 2023. The diagnosis of POUR was made in a woman who completely failed to void despite a full bladder or, one who had post-void residual (PVR) > 150 ml within 10 min of voiding. Return to normal voiding was considered if a patient with POUR had two consecutive PVRs of ≤ 150 ml. Descriptive analyses and multivariable logistic regression were performed to determine risk factors for POUR.

**Results:**

A total of 153 participants were enrolled in this study with a mean age of 35.9 (SD ± 10.8) years. The incidence of POUR was 19.6% (30/153, 95% CI 14.02–26.7), and the median time to normal voiding for these patients was 42.4 h (range 24–72). Risk factors for POUR included repeat perineal tear surgery (RR = 4.24; 95% CI 1.16–15.52; *p* = 0.029) and early urinary catheter removal (RR = 2.89; 95% CI 1.09–7.67; *p* = 0.033).

**Conclusion:**

Post-operative urinary retention following surgery for perineal tears is common. The time to return to normal voiding in patients with POUR is short. Women having repeat perineal tear surgery and those in whom the urinary catheter is removed early were more likely to experience POUR. Delayed urinary catheter removal could be considered, especially in patients undergoing repeat perineal tear surgery.

## Introduction

Post-operative urinary retention (POUR) is the inability to void despite a full bladder in the post-operative period often characterized by impaired bladder emptying with a rise in the volume of retained urine [1]. The prevalence of POUR varies with type of surgery. Rates of 15–45% have been reported following pelvic surgery especially for urine incontinence and pelvic organ prolapse [2–5].

Post-operative urinary retention could result from the effects of surgery or drugs used in the peri-operative period. Effects of surgery such as tissue edema, inflammation, denervation, and post-operative pain alter the bladder physiology, leading to POUR [6]. Drugs used in the peri-operative period such as anesthetics and analgesics, especially opioids, can lead to POUR through relaxation of the detrusor muscle or suppression of the central and peripheral micturition reflexes [7, 8]. There are various ways of determining POUR but the standard is measurement of post-void residual (PVR) either by in-and-out catheterization or by bladder ultrasound. There is currently no standard PVR to define POUR, but previous studies have used cutoffs of > 100 ml [9], > 150 ml [10], or > 200 ml [4, 11].

Undiagnosed or untreated POUR can lead to significant morbidities such as discomfort, urinary tract infection, prolonged bladder distension with associated detrusor injury, ureteric reflux resulting into renal dysfunction, and may even cause breakdown of the repair [9, 12, 13]. POUR also causes the patient worry and anxiety [14].

Previously reported risk factors for POUR following pelvic floor surgeries include lower body mass index, previous urinary incontinence surgery, age > 50 years, anterior colporrhaphy, high pre-operative PVR, and a bladder volume ≥ 270 ml in the immediate post-anesthetic period [15–18]. However, most of these studies concern POUR following pelvic organ prolapse and urinary incontinence surgery [15, 17–19]. There is therefore a paucity of data on POUR following surgery for perineal tears, especially in the low-income countries, yet this is one of the most common surgeries in this setting. We hypothesized that women who have had perineal tear surgery are likely to suffer POUR because of the presence of the various etiologies such as tissue edema, surgical site hematoma, and surgical site pain. Furthermore, predicting POUR is critical for early diagnosis and successful management. Therefore, in this study, we aimed to determine the incidence of, and risk factors for POUR following surgery for perineal tears, and to determine the time taken to attain normal voiding at a tertiary referral hospital in Southwestern Uganda.

## Materials and Methods

### Study Setting and Study Design

This study was a prospective single-arm cohort of women with obstetric perineal tears who underwent repair at our tertiary facility from January 2022 to December 2023.

### Study Population

We enrolled women scheduled for repair of old obstetric perineal tears (≥ 3 months after child birth) who consented to take part in the study. The perineal tears were graded into first, second, third, and fourth degrees [20, 21]. Those eligible for surgery were women with second-, third-, and fourth-degree perineal tears. We excluded women who were scheduled to have other pelvic floor surgeries such as prolapse or urine incontinence at the same time as the perineal repair. Surgical eligibility was evaluated by the clinical care team.

### Surgery

The participants underwent surgery for the perineal tears after obtaining informed consent. Surgery was dependent on the degree of perineal tear. Perineorrhaphy was performed in those with second-degree perineal tears. In those who had third- and fourth-degree perineal tears, perineorrhaphy plus sphincteroplasty was the surgery performed. All surgeries were performed under spinal anesthesia, by a team of subspecialty surgeons (certified urogynecologists and urogynecology fellows), as part of the routine management of perineal tears at the hospital.

### Post-Operative Care

Post-operative care was the same for all the participants. They all had an indwelling Foley catheter inserted at the end of the surgery. This catheter was either removed within 24 h after surgery (early catheter removal) or 24 h post-surgery (delayed catheter removal). This was at the discretion of the attending surgeon. In addition, antibiotics and other elements of post-operative care, such as non-opioid analgesia (paracetamol and nonsteroidal anti-inflammatory drugs), fluid administration, and ambulation, were given to all the participants, which is the routine care protocol in the unit.

### Diagnosis of POUR

All the participants underwent a passive voiding trial. Once the Foley catheter had been removed during the routine post-operative care, the participants were given time to fill their bladders and void when they felt the urge. A PVR was then measured within 10 min after voiding. The diagnosis of POUR was made in a participant who completely failed to void, despite a full bladder, or one that had PVR > 150 ml within 10 min after voiding [10]. PVR was measured with in-and-out catheterization, which is the usual protocol for the hospital.

### Management of POUR and Time to Normal Voiding

In women who developed POUR, bladder decompression was done by reinserting an indwelling catheter. The catheter was kept in for 24 h, after which a passive voiding trial was repeated and PVR measured. A participant was considered to have passed the voiding trial (normal voiding) if she had two consecutive PVRs of ≤ 150 ml. If any of the PVRs was > 150 ml, an indwelling catheter was reinserted for another 24 h until the voiding trial was passed. The time to achieve normal voiding was that from diagnosis of POUR to the second normal PVR.

### Data Collection

A data capture tool was used to collect information on the baseline characteristics of the study participants, intra-operative findings, and the post-operative information. The baseline characteristics included:

1. Sociodemographic characteristics (age, marital status)
2. Gynecological history (parity, menopausal status, duration with the perineal tear and history of previous perineal tear surgery)
3. Degree of perineal tear (second-, third-, and fourth-degree)

The intra-operative information that was collected included:

1. Surgical procedure performed
2. Length of surgery in minutes
3. Intra-operative complications (hemorrhage that required transfusion)

Post-operative information collected included:

1. Post-operative complications (surgical site hematoma, constipation, and wound infection)
2. Timing of catheter removal (early or delayed catheter removal)
3. Outcome (POUR and no POUR)
4. Time to normal voiding in hours for those who had POUR

The data capture form was filled out by the trained research assistants (nurses and surgeons).

### Statistical Analysis

Data were entered into REDCap and exported to Stata 13 (StataCorp, College Station, TX, USA) for analysis. Categorical data were presented as frequencies. The incidence of POUR was determined by dividing the number of women who had POUR by the total number of women who underwent surgery for perineal tears and expressed as a percentage. Differences in demographic and clinical characteristics comparing those with POUR and those without POUR were assessed using Chi-squared test or Fisher’s exact test.

To determine the risk factors for POUR, bivariate and multivariate analyses were performed using log binomial regression analysis. Risk ratios (RR) and their corresponding 95% confidence intervals (CIs) were reported as the measures of association. Factors with a *p* value < 0.2 at bivariate analysis were included in the final multivariate model to determine the adjusted risk factors for POUR. A *p* value < 0.05 was considered statistically significant.

## Results

A total of 153 women were enrolled into this study. Of these, 30 developed POUR. The incidence of POUR was 19.6% (95% CI 14.02–26.7).

The baseline participant characteristics are shown in Table 1. The mean age of the participants was 35.9 (SD ± 10.8) years. The majority were of parity ≥ 2 (*n* = 131, 85.6%), pre-menopausal (*n* = 140, 91.5%) and had had the perineal tear for > 1 year (*n* = 126, 82.4%). The majority of the participants had fourth-degree perineal tears (*n* = 114, 74.5%). All operations were performed under spinal anesthesia. The commonest surgery performed was a perineorrhaphy with sphincteroplasty and in 72.5% of the participants, this was a primary surgery. Sixteen women (10.5%) experienced post-operative complications, with the main complications being surgical site hematoma (*n* = 8, 5.2%) and constipation that required rectal disimpaction (*n* = 8, 5.2%). The majority of participants (*n* = 28, 93.3%) had complete POUR (unable to void at all). The mean PVR was 933.3 ml (SD ± 238.3). The median time to normal voiding in the participants who suffered from POUR was 42.4 h (range 24–72 h). None of the women required further catheterization after 72 h and therefore no one was discharged with a catheter, as shown in Table 2.

**Table 1:** Demographic, clinical, and peri-operative characteristics of the study participants by post-operative urinary retention (*POUR*) status

Characteristic	Total cohort (*N* = 153)	POUR		
		No (*n* = 123)	Yes (*n* = 30)	*p* value
Age in years, mean (SD)	36.0 (10.80)	35.7 (10.96)	37.27 (10.20)	0.47
Parity, *n* (%)				0.054
Primipara (1)	22 (14.4)	21 (17.1)	1 (3.3)	
Multipara (≥ II)	131 (85.6)	102 (82.9)	29 (96.7)	
Reached menopause, *n* (%)	13 (8.5)	11 (8.9)	2 (6.7)	0.69
Duration with tear, *n* (%)				0.22
<1 year	27 (17.6)	24 (19.5)	3 (10.0)	
≥1 year	126 (82.4)	99 (80.5)	27 (90.0)	
Degree of perineal tear, *n* (%)				0.2
Second degree	12 (7.8)	12 (9.8)	0 (0.0)	
Third degree	27 (17.6)	21 (17.1)	6 (20.0)	
Fourth degree	114 (74.5)	90 (73.2)	24 (80.0)	
Type of perineal surgery, *n* (%)				0.075
Perineorrhaphy alone	12 (7.8)	12 (9.8)	0 (0.0)	
Perineorrhaphy with sphincteroplasty	141 (92.2)	111 (90.2)	30 (100.0)	
Cadre of surgeon, *n* (%)				0.110
Urogynecology Fellow	67 (43.8)	50 (40.7)	17 (56.7)	
Urogynecologist	86 (56.2)	73 (59.3)	13 (43.3)	
Duration of surgery, *n* (%)				0.30
<1 h	35 (22.9)	26 (21.1)	9 (30)	
≥1 h	118(77.1)	97 (78.9)	21 (70)	
Early post-operative complications, *n* (%)				<0.001
Constipation	8 (5.2)	3 (2.4)	5 (16.7)	
Surgical site hematoma	8 (5.2)	2 (1.6)	6 (20.0)	
None	137 (89.5)	118 (95.9)	19 (63.3)

**Table 2:** Description of post-operative urinary retention (*POUR*) among women with surgery for perineal tears

Characteristic of POUR		Total with POUR, *N* = 30	
		Frequency	Percentage
Nature of POUR			
Complete (completely unable to void)		28	93.3
Partial (voids but PVR > 150 ml)		2	6.7
Mean residual volume, ml (SD)			
933.3 (±238.3)			
Time to normal voiding, h (recovery from POUR)			
24		11	37
48		15	50
72		4	13

In the multivariate analysis, repeat perineal tear surgery (aRR = 4.24; 95%CI 1.16–15.52; *p* = 0.029) and early removal of the urinary catheter after surgery (aRR = 2.89; 95% CI: 1.09–7.67; *p* = 0.033) were the risk factors for POUR following surgery for perineal tears, as shown in Table 3.

**Table 3:** Risk factors for post-operative urinary retention (*POUR*) among women with surgery for perineal tears

Characteristic	POUR				Multivariable analysis	
	No (*N* = 123)	Yes (*N* = 30)	Crude RR (95% CI)	*p* value	Adjusted RR (95% CI)	*p* value
Age (years)						
<50	107 (87.0%)	28 (93.3%)	Ref		Ref	
≥50	16 (13.0%)	2 (6.7%)	0.48 (0.10–2.20)	0.343	0.44 (0.08–2.35)	0.337
Parity						
Primipara (1)	21 (17.1%)	1 (3.3%)	Ref		Ref	
Multipara (≥ II)	102 (82.9%)	29 (96.7%)	5.97 (0.77–46.28)	0.087	10.45 (0.99–110.43)	0.051
Duration of surgery (h)						
<1	26 (21.1%)	9 (30.0%)	Ref		Ref	
≥1	97 (78.9%)	21 (70.0%)	0.63 (0.26–1.53)	0.303	0.63 (0.21–1.87)	0.408
Nature of surgery						
Primary/index	107 (87.0%)	24 (80.0%)	Ref		Ref	
Repeat (≥1)	16 (13.0%)	6 (20.0%)	1.49 (0.69–3.22)	0.313	4.24 (1.16,15.52)	0.029*
Timing of urinary catheter removal post-surgery						
Within 24 h	35 (28.5%)	18 (60.0%)	2.83 (1.48,5.42)	0.002*	2.89 (1.09,7.67)	0.033*
After 24 h	88 (71.5%)	12 (40.0%)	Ref		Ref	

*CI* confidence interval, *RR* risk ratio, *Ref* reference category

*p < 0.05

## Discussion

This study determined the incidence of POUR following surgery for old perineal tears and the risk factors. We found an incidence of 19.6% and the risk factors were repeat perineal tear surgery and early urinary catheter removal after surgery.

We found no comparative studies on POUR following perineal tear surgery. Most of the studies are on POUR following surgery for pelvic organ prolapse and urinary incontinence. The incidence of POUR in this study is lower than that in other studies performed in Indonesia and the Netherlands that both reported a POUR rate of 29% after pelvic organ prolapse surgery [4, 11]. The contrast could be due to the difference in etiology of POUR following surgery for the two conditions. POP surgeries such as anterior repair can cause damage to small peripheral nerve endings important for bladder sensation, resulting in bladder dysfunction and leading to retention [9]. Furthermore, the study by Hakvoort et al. [4] also involved women who underwent urinary incontinence surgeries such as a Kelly plication procedure, known to increase the risk for retention [18, 19, 22]. In addition, pelvic prolapse surgery can lead to elevation of the bladder neck and edema of the paravesical tissues that may lead to POUR [15]. However, none of that occurs in perineal tear surgery and this could explain the lower incidence of POUR in our study.

The median time to recovery of POUR (normal voiding) in our study of 42.4 (range 24–72) hours was shorter than that found in other studies [4, 17]. Sokol et al. [17] reported a median time to adequate voiding of 5 days (range 0–32) following surgery for urinary incontinence using TVT, and it was even longer (8 days) where additional pelvic organ prolapse treatment was carried out. Another study found that 34% of patients needed re-catheterization for more than 72 h after surgery [4]. The shorter recovery time of POUR after perineal surgeries compared with that in other pelvic floor operations is probably due to the differences in etiology. POUR following perineal tear surgery, which is mainly due to post-operative pain and drugs (anesthetics and opioid analgesics) [7, 8], resolves faster than that following surgery for prolapse and incontinence that is due to damage to bladder innervation [9].

One of the risk factors for POUR in this study was repeat perineal tear surgery. This is similar to what was found in other studies [17, 19], where the risk of POUR was higher in patients with a history of previous incontinence surgery. The risk of POUR in patients who have undergone repeat surgeries could be multifactorial. Repeat surgeries could take longer than the primary ones and hence longer hours under anesthesia, and they may have more intra-operative bleeding, more hematomas post-operatively, and more post-operative pain, all of which are known risks for POUR [4, 16, 23, 24].

Post-operative urinary retention was more likely to occur in women who had early removal of the urinary catheter after surgery. This is similar to a study by Hakvoort et al. in which 40% of patients who had early catheter removal needed re-catheterization, compared with 8% in whom the catheter was removed after 5 days [4]. However, other studies found that early catheter removal was not associated with high rates of POUR [25–27]. This contrast could be due to the design in the studies. All the above studies were randomized controlled clinical trials, unlike our study where catheter removal was at the discretion of the attending surgeon, which could have introduced a bias. However, in all the contrasting studies, the surgery was either for urinary incontinence or for pelvic organ prolapse. None was to treat perineal tear surgery. Therefore, randomized controlled clinical trials among perineal tear surgeries comparing rates of POUR between early and delayed catheter removal need to be conducted to clarify this issue.

In this study, POUR was not associated with increasing age, parity, BMI, menopausal status, type of anesthesia, type of surgery, and duration of surgery, as reported in other studies [4, 18, 19, 22, 28]. All patients in this study were operated upon under spinal anesthesia and only one type of surgery (perineal repair) was performed. Duration under anesthesia has been found to be a risk factor for POUR, with longer anesthesia time carrying a higher risk [7, 8]. Both general and spinal anesthesia can lead to POUR through relaxation of the detrusor muscle or suppression of the central and peripheral micturition reflexes [7, 8]. Also, the type of perineal tear surgery could be a risk factor for POUR, with fourth-degree perineal tears that involve extensive dissection carrying a higher risk than second-degree perineal tears. Surgery for fourth-degree perineal tears could take longer than that for second-degree tears and hence longer anesthesia hours, more intra-operative bleeding, more hematomas post-operatively, and more post-operative pain, all of which are known risks for POUR [4, 16, 23, 24]. However, these were not assessed in our study.

To the best of our knowledge, this is one of the first studies to describe the challenge of POUR following surgery for old perineal tears especially in low-resource settings where the surgery is fairly common. Also, the data were collected prospectively, hence eliminating the multiple shortcomings of retrospective studies, such as missing data. The PVR was assessed using the in-and-out catheter method, which has been shown to provide more accurate and precise PVR data than bladder ultrasound. Our study, however, had some limitations. First and foremost, this was a single-arm cohort without a comparative group. Therefore, we were unable to compare the rates of POUR following perineal tear surgery with those of other pelvic floor surgeries such as prolapse and incontinence. Second, there is no standard PVR cutoff for the diagnosis of POUR and therefore, the 150 ml that we used in this study could have led to an over-estimation of POUR. Third, pre-operative PVRs were not assessed and we are therefore not certain if the patients who developed POUR did not have underlying voiding dysfunction prior to surgery.

## Conclusion

Post-operative urinary retention following surgery for old perineal tears is common, occurring in about 2 in every 10 women. The recovery time for POUR after perineal tear surgery is short. Women having repeat perineal tear surgery and those with early removal of the urinary catheter are more likely to experience POUR. Further studies are needed to explore the difference in rates of POUR between patients who have early catheter removal and those with delayed catheter removal following perineal tear surgery.

## Data Availability

The datasets generated and analyzed during the study are available from the corresponding author on request.
